# How the Law Regards the Loss of One Eye

**Published:** 1916-08-05

**Authors:** 


### How the Law Regards the Loss of One Eye.

It is a much debated question whether the Court, in considering what is or is not suitable employment for a one-eyed workman in cases where the employer wishes to cease payment of the man's compensation money, ought to have regard to the specially serious consequences to him of the possible risk of an accident which may result in his becoming completely blind through an injury to his remaining eye. The judges have been far from unanimous in their opinion, their divergent views being well illustrated by the opinions respectively given in one of the cases by Lords Justices Buckley and Fletcher Moulton. The former said, "I do not myself accept that it would be a good reason to assign that having lost one eye the loss of the second would be of greater import to the man in question than to any other man. Of course it would be, but I do not think that is relevant upon the question of suitability." On the other hand, the latter judge remarked, "In my opinion you cannot consider an employment suitable that a reasonable, careful man desirous of earning his living is entitled to reject because it exposes him to risks so serious in their consequences that he feels he is not doing his duty to himself and his family in encountering them." We regret to see that the more recent judicial opinions are inclining in the direction of Lord Justice Fletcher Moulton's view.

